# Ablação por Campo Pulsado para Fibrilação Atrial: Experiência Inicial no Brasil

**DOI:** 10.36660/abc.20250893

**Published:** 2026-03-03

**Authors:** Gabriela Miana de Mattos Paixão, Isadora Cristine Reis Sguizzato Bozzi

**Affiliations:** 1 Universidade Federal de Minas Gerais Belo Horizonte MG Brasil Universidade Federal de Minas Gerais, Belo Horizonte, MG – Brasil

**Keywords:** Arritmias Cardíacas, Fibrilação Atrial, Terapia de Eletroporação Irreversível

A fibrilação atrial (FA) é a arritmia cardíaca sustentada mais comum na prática clínica e representa um fardo significativo para a saúde pública, pois está associada de forma independente à redução da qualidade de vida, ao aumento do risco de acidente vascular cerebral e à mortalidade por todas as causas.^1^ A incidência e a prevalência da FA estão aumentando em todo o mundo, impulsionadas principalmente pelo envelhecimento da população e pela crescente prevalência de comorbidades cardiovasculares. Em conjunto, esses achados explicam o impacto econômico da FA e seu papel como um desafio crescente para a saúde pública.

A ablação por cateter é uma estratégia bem estabelecida para o controle do ritmo na FA, tendo o isolamento das veias pulmonares como componente central, desde que os fatores desencadeantes relacionados às veias pulmonares foram identificados.^1^ Ao longo de mais de duas décadas, os avanços no mapeamento eletroanatômico, na tecnologia de cateteres e na aplicação de energia melhoraram a segurança e os resultados a longo prazo da ablação por radiofrequência e crioablação. Apesar desses avanços, o sucesso em um único procedimento permanece limitado e podem ocorrer complicações relacionadas a lesões térmicas colaterais. A ablação por campo pulsado (PFA) surgiu recentemente como uma nova modalidade de ablação não térmica baseada na eletroporação, induzida por pulsos elétricos rápidos que ablacionam seletivamente o tecido miocárdico, minimizando lesões em estruturas adjacentes, como o esôfago e o nervo frênico. Estudos clínicos iniciais e registros do mundo real demonstraram um perfil de segurança favorável e eficácia comparável à ablação térmica.^2-4^ No entanto, a otimização de protocolos e a obtenção de dados robustos sobre os resultados a longo prazo continuam sendo necessárias, especialmente em países de baixa e média renda, como o Brasil.

O primeiro sistema PFA foi aprovado pela Agência Nacional de Vigilância Sanitária (ANVISA) apenas em abril de 2024. Portanto, seu uso no Brasil é recente, e a literatura publicada disponível pode diferir dos resultados brasileiros devido a diferenças na infraestrutura de saúde, experiência do operador e demografia da população. Para preencher essa lacuna, Ávila et al.^5^ realizaram o primeiro registro prospectivo brasileiro de 394 procedimentos PFA consecutivos realizados entre 18 de junho de 2024 e 29 de abril de 2025, em 60 centros participantes.

A população estudada tinha uma idade média de 65 ± 12 anos, sendo 64% do sexo masculino. A FA paroxística foi a principal indicação em 62% dos pacientes, seguida pela FA persistente em 34%. O isolamento exclusivo das veias pulmonares foi o conjunto de lesões predominante (54%), e o isolamento da parede posterior foi realizado em 168 pacientes (43%), seja durante o procedimento inicial ou em uma reintervenção. O número médio de aplicações foi de 51 ± 16, com um tempo médio total de procedimento de 113 ± 67 minutos. A anestesia geral foi realizada em 98% dos casos. A ablação guiada por fluoroscopia foi utilizada na maioria dos procedimentos, sendo que 75% dos procedimentos utilizaram mapeamento eletroanatômico 3D. Os procedimentos sem fluoroscopia (12%) apresentaram um número significativamente maior de aplicações (74±22 vs. 48±12, p<0,001), tempos de ablação e de procedimento total mais longos (53±14 vs. 40±25 minutos, p=0,002 e 145±37 vs. 106±67 minutos, p<0,001, respectivamente) em comparação com os procedimentos que utilizaram fluoroscopia. Nenhuma complicação grave foi relatada nesta série inicial.

O estudo também comparou os três centros com maior volume de procedimentos (Grupo I = 139 pacientes – 35%) com o restante do Grupo II (255 pacientes – 65%). Os centros com alto volume de procedimentos apresentaram tempos de procedimento e ablação significativamente menores (82 ± 27 vs. 131 ± 76 minutos; p < 0,001 e 33 ± 14 vs. 50 ± 33 minutos; p < 0,001, respectivamente), além de menos lesões adicionais após o remapeamento (7% vs. 30%; p < 0,001), sugerindo uma curva de aprendizado rápida.

Esses achados fornecem resultados encorajadores, consistentes com outras pesquisas internacionais,^3,6^ e oferecem novas perspectivas relevantes sobre o uso atual da ablação por radiofrequência para FA no Brasil. O procedimento demonstra alta segurança aguda e uma curva de aprendizado curta, como evidenciado pela eficiência de operadores com grande volume de procedimentos. Entretanto, esta é uma revisão importante, porém preliminar. Os autores reconhecem que a falta de acompanhamento é uma limitação que deve ser abordada em pesquisas futuras por meio de estudos de desfecho em longo prazo. Além disso, análises de custo-efetividade também são necessárias para avaliar a viabilidade da incorporação dessa tecnologia aos planos de saúde e ao sistema público de saúde brasileiros.
