# Supplementary material for: Ablação por Campo Pulsado para Fibrilação Atrial: Experiência Inicial no Brasil
Source: Arq Bras Cardiol. 2026 Mar 3;123(1):e20250893. [Article in Portuguese] doi: 10.36660/abc.20250893 (PMC13127810; doi:10.36660/abc.20250893)
Supplement: Translation

# Translation: Pulsed Field Ablation for Atrial Fibrillation: Early Experience in Brazil

**DOI:** 10.36660/abc.20250893i

**Authors:** Gabriela Miana de Mattos Paixão, Isadora Cristine Reis Sguizzato Bozzi

**Affiliations:** 1 Belo Horizonte MG Brazil Universidade Federal de Minas Gerais, Belo Horizonte, MG – Brazil

**Keywords:** Cardiac Arrhythmias, Atrial Fibrillation, Irreversible Electroporation Therapy

Atrial fibrillation (AF) is the most common sustained cardiac arrhythmia in clinical practice and represents a significant public health burden because it is independently associated with impaired quality of life, increased risk of stroke, and all-cause mortality^(1)^. The incidence and prevalence of AF are growing worldwide, driven mainly by population aging and the rising prevalence of cardiovascular comorbidities. Together, these findings explain the economic impact of AF and its role as a growing healthcare challenge.

Catheter ablation is a well-established rhythm-control strategy for AF, with pulmonary vein isolation as its central component, since pulmonary vein–related triggers were recognized.^1^ Over more than two decades, advances in electroanatomic mapping, catheter technology, and energy delivery have improved the safety and long-term outcomes of radiofrequency and cryoballoon ablation. Despite these advances, single-procedure success remains limited, and complications related to collateral thermal injury can occur. Pulsed field ablation (PFA) has recently emerged as a novel, nonthermal ablation modality based on electroporation, induced by rapid electrical pulses that selectively ablate myocardial tissue while minimizing injury to adjacent structures such as the esophagus and phrenic nerve. Early clinical studies and real-world registries have demonstrated a favorable safety profile and efficacy comparable to thermal ablation.^2-4^ However, optimizing protocols and obtaining robust long-term outcome data remain necessary, especially in low- and middle-income countries such as Brazil.

The first PFA system was approved by the Brazilian Health Regulatory Agency (ANVISA) only in April 2024. Hence, its use in Brazil is recent, and the available published literature may differ from Brazilian results due to differences in healthcare infrastructure, operator experience, and population demographics. To fill this gap, Avila et al.^5^ conducted the first Brazilian prospective registry of 394 consecutive PFA procedures performed between June 18, 2024, and April 29, 2025, at 60 participating centers.

The study population had a mean age of 65 ± 12 years, and 64% were male. Paroxysmal AF was the primary indication in 62% of patients, followed by persistent AF in 34%. Pulmonary vein isolation-only was the dominant lesion set (54%), and posterior wall isolation was performed in 168 patients (43%) either during the index or a redo procedure. The mean number of applications was 51 ± 16, with a total mean procedure time of 113 ± 67 minutes. General anesthesia was performed in 98% of the cases. Fluoroscopy-guided ablation was used in most procedures, with 75% of procedures using 3D electroanatomical mapping. Fluoroless procedures (12%) had a significantly higher number of applications (74±22 vs. 48±12, p<0.001), longer ablation and total procedure times (53±14 vs. 40±25 minutes, p=0.002 and 145±37 vs. 106±67 minutes, p<0.001, respectively) as compared to procedures using fluoroscopy. No major complications were reported during this initial series.

The study also compared the three highest-volume operators (Group I = 139 patients – 35%) with the remaining Group II (255 patients – 65%). High-volume centers had significantly shorter procedure and ablation times (82 ± 27 vs. 131 ± 76 minutes; p <0.001 and 33 ± 14 vs. 50 ± 33 minutes; p <0.001, respectively), alongside fewer additional lesions after remapping (7% vs. 30%; p<0.001), suggesting a rapid learning curve.

These findings provide encouraging results consistent with other international research^3,6^ and offer new, relevant insights into the current use of PFA for AF in Brazil. It demonstrates high acute safety and a short learning curve, as evidenced by the efficiency of high-volume operators. However, this is an important, but preliminary overview. The authors acknowledge that the lack of follow-up is a limitation, which should be addressed in future research through long-term outcome studies. Furthermore, cost-effectiveness analyses are also warranted to evaluate the feasibility of incorporating this technology into Brazilian health insurance plans and the public health system.
